# MicroRNA-103 Promotes Colorectal Cancer by Targeting Tumor Suppressor DICER and PTEN

**DOI:** 10.3390/ijms15058458

**Published:** 2014-05-13

**Authors:** Li Geng, Bing Sun, Bo Gao, Zheng Wang, Cheng Quan, Feng Wei, Xue-Dong Fang

**Affiliations:** 1 Department of General Surgery, the Second Hospital of Jilin University, Changchun 130041, Jilin, China; E-Mails: gengli79@gmail.com (L.G.); gb2664402136@gmail.com (B.G.); wangzheng19690101@gmail.com (Z.W.); quancheng46@gmail.com (C.Q.); 2 Department of Neurology, Changchun Central Hospital, Changchun 130041, Jilin, China; E-Mail: sunfangqi78@gmail.com; 3 Department of Hepatobiliary and Pancreas Surgery, the First Hospital, Jilin University, Changchun 130021, Jilin, China

**Keywords:** miR-103, *DICER*, *PTEN*, proliferation, migration, colorectal cancer

## Abstract

MicroRNAs (miRNAs) are a class of small, noncoding RNAs that act as key regulators in various physiological and pathological processes. However, the regulatory mechanisms for miRNAs in colorectal cancer remain largely unknown. Here, we found that miR-103 is up-regulated in colorectal cancer and its overexpression is closely associated with tumor proliferation and migration. In addition, repressing the expression of miR-103 apparently inhibits colorectal cancer cell proliferation and migration *in vitro* and HCT-116 xenograft tumor growth *in vivo*. Subsequent software analysis and dual-luciferase reporter assay identified two tumor suppressor genes *DICER* and *PTEN* as direct targets of miR-103, and up-regulation of *DICER* and *PTEN* obtained similar results to that occurred in the silencing of miR-103. In addition, restoration of *DICER* and *PTEN* can inhibit miR-103-induced colorectal cancer cell proliferation and migration. Our data collectively demonstrate that miR-103 is an oncogene miRNA that promotes colorectal cancer proliferation and migration through down-regulation of the tumor suppressor genes *DICER* and *PTEN*. Thus, miR-103 may represent a new potential diagnostic and therapeutic target for colorectal cancer treatment.

## 1. Introduction

Colorectal cancer is the third most commonly diagnosed cancer worldwide, especially in developed countries [1,2], with symptoms like rectal bleeding and anemia sometimes associated with weight loss and changes in bowel habits [3,4]. It has been reported that colorectal cancer is usually derived from uncontrolled cell growth in the intestine [5,6] and most occurs owing to lifestyle and increasing age [2]. Colorectal cancers which confined within the wall of colon are usually curable by chemotherapy or surgery, while cancers with strong metastasis activity are often lethal. Therefore, early diagnosis usually means life-saving and more and more attention has been paid to find meaningful diagnostic targets for colorectal cancer [7].

MicroRNAs (miRNAs) are small non-coding RNAs that post-transcriptionally modulate gene expression by repressing translation or accelerating mRNA degradation [8,9]. They are highly conserved among species and play important roles in various physiological and pathological processes including developmental abnormalities, autoimmune diseases and cancers [10–12]. Several aberrant expressed miRNAs have been reported to be involved in tumorigenesis as either oncogenes or tumor suppressors [13,14].

In order to investigate the roles of miRNAs that played in colorectal cancer, we did a microarray and we found that miR-103 is one of the most significantly up-regulated miRNA in colorectal cancers and the proliferation of colorectal cancer cells is apparently repressed when miR-103 was silenced. We further identified *DICER* and *PTEN*, two critical tumor repressor genes, as target genes of miR-103. Up-regulating *DICER* or *PTEN* by transfecting with pcDNA-3.1-*DICER*/pcDNA-3.1-*PTEN* plasmids repressed cancer cell proliferation and migration. When used in combination with miR-103, both *DICER* and *PTEN* apparently abrogated the effect of miR-103 on colorectal cancer cell proliferation and migration. To verify all these *in vitro* data, xenograft models were generated by HCT-116 cells with up-regulated or down-regulated miR-103 levels (transfected by lenti-miR-103 or agomir-miR-103, respectively), results showed that up-regulation of miR-103 significantly promoted, whereas down-regulation of miR-103 inhibited the growth of xenografts *in vivo*. Collectively, our study demonstrated that miR-103 may represent a new potential therapeutic target for colorectal carcinoma treatment by targeting *DICER* and *PTEN*.

## 2. Results and Discussion

### 2.1. miR-103 Is Up-Regulated in Colorectal Cancer Patients

**Figure 1.**
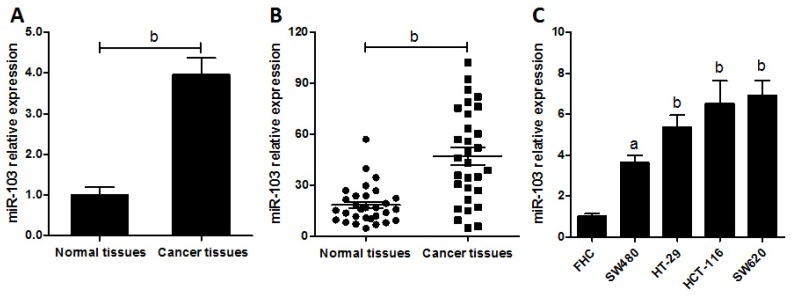
miR-103 is up-regulated in colorectal cancer. (**A**) RNAs were isolated from the colorectal cancer tissues and the matched normal control tissues, and miRNA expression profiles were determined by miRNA microarray. miR-103 expression was normalized to control of random sequences of a similar size. One representative of two experiments is shown; (**B**) The expression level of mature miR-103 in colorectal cancer tissues and their matched normal tissues were determined by real-time polymerase chain reaction (PCR); and (**C**) The expression level of miR-103 in four colorectal cancer cell lines and normal colonic cells were checked by real-time PCR. Data are representative of three experiments. Error bars represent as mean ± SD. ^a^ *p* < 0.05; ^b^ *p* < 0.01 *vs.* normal control.

According to our miRNA array data of five-pair of samples of colorectal cancer patients, miR-103 was found significantly overexpressed in carcinoma tissues compared with their matched normal tissues (Figure 1A). To confirm this, miR-103 levels were measured in 30 cases of colorectal carcinoma tissues by real-time polymerase chain reaction (RT-PCR). Results showed that miR-103 was apparently up-regulated in cancer tissues compared with normal controls (Figure 1B, *p* < 0.01). To further identify that this phenomenon is consistent and common in colorectal cancer cell lines, the expression of miR-103 in a panel of four colorectal carcinoma cell lines and a normal colonic cell line was detected by RT-PCR. As shown in Figure 1C, the expression of miR-103 was significantly higher in cancer cells than that in normal control. These data uncovered that the expression of miR-103 was universally up-regulated in colorectal cancer cells and patients, indicating that increased miR-103 expression might contribute to tumor malignant phenotype and tumor development.

### 2.2. miR-103 Directly Promotes Colorectal Carcinoma Cell Proliferation and Migration

**Figure 2.**
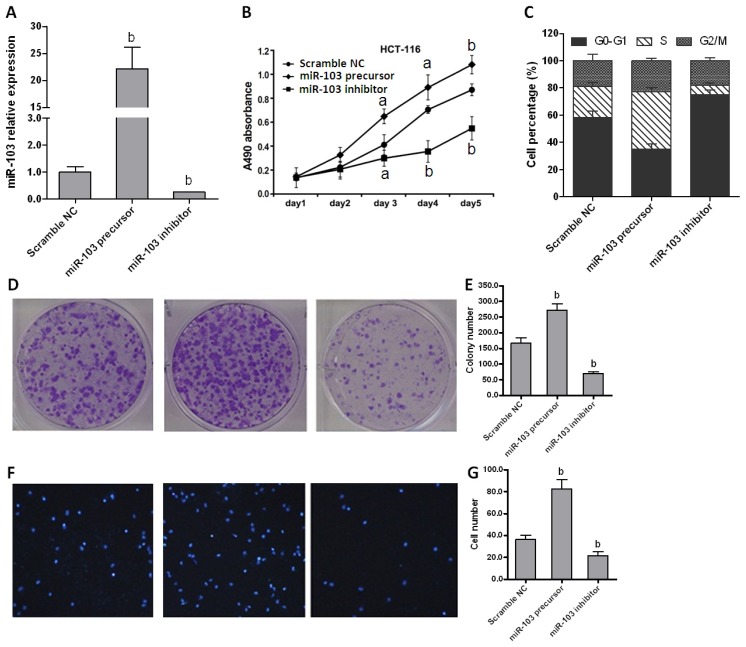
miR-103 affects colorectal carcinoma cell proliferation and cell cycle *in vitro*. (**A**) Expression of miR-103 in HCT-116 cells transfected with scramble negative control (NC), miR-103 precursor or inhibitor was quantified by real-time PCR; (**B**) Proliferation rates of HCT-116 cells were detected by 3-(4,5-dimethylthiazol-2-yl)-2,5-diphenyltetrazolium bromide (MTT) assay; (**C**) Cell cycle distributions of HCT-116 cells were analyzed by flow cytometry; (**D**) HCT-116 cells were cultured for 10 days after transfection and cell viability was analyzed by colony formation assay; (**E**) analysis results of **D**; (**F**) Transwell assay was used to detect cell migration (40× magnification). Data are representative of three experiments; and (**G**) analysis results of **F**. Data are representative of three experiments. Error bars represent as mean ± SD. ^a^ *p* < 0.05; ^b^ *p* < 0.01 *vs.* scramble NC.

To investigate the role of miR-103 in colorectal cancer cell proliferation, miR-103 was over-expressed or repressed in HCT-116 cells by transfection with miR-103 precursor or inhibitor (Figure 2A). 3-(4,5-Dimethylthiazol-2-yl)-2,5-diphenyltetrazolium bromide (MTT) assay showed that over-expression of miR-103 accelerated cell proliferation (*p* < 0.01). Conversely, down-regulated miR-103 delayed cell proliferation in comparison with control group (Figure 2B). Similar data were also obtained in other three cell lines (data not shown). These results indicated that miR-103 plays an important role in regulating the proliferation of colorectal cancer cells. Then, flow cytometry was performed to evaluate whether miR-103 affected cell cycle distribution. Cells transfected with miR-103 precursor represented a significant reduction of cells proportion in G0/G1 phases and an accumulation in S phase, whereas miR-103 inhibitor transfection caused an accumulation of cells in G0/G1 phases and a strong reduction in S phase compared with the scramble control (Figure 2C). These data indicated that miR-103 promotes cell proliferation at least in part by inducing cell cycle release from G0/G1 phases.

Then colony formation assay was carried out to evaluate the effect of miR-103 on the colony-forming ability of cancer cells. As shown in Figure 2D,E, the colony numbers significantly increased in cells transfected with miR-103 precursor when compared with the control (*p* < 0.01), whereas cells transfected miR-103 inhibitor displayed dramatically impaired colony growth ability. Finally, a transwell assay was performed to examine the potential role of miR-103 in tumor migration. Figure 2F,G showed that HCT-116 transfected with miR-103 precursor exhibited more invasion ability compared with cells transfected with miR-103 inhibitor (*p* < 0.01). Collectively, all these data demonstrated that miR-103 effectively promotes colorectal carcinoma by accelerating cell proliferation and migration.

### 2.3. DICER and PTEN Are Direct Target Genes of miR-103

**Figure 3.**
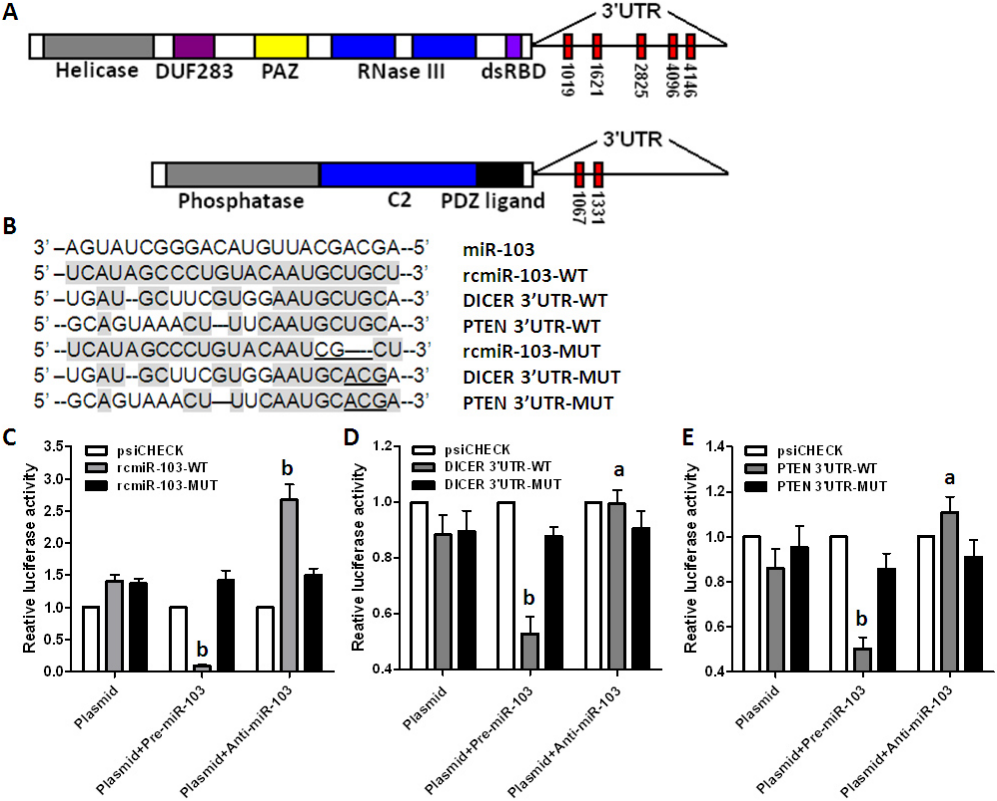
*DICER* and *PTEN* are direct target genes of miR-103. (**A**) Schematic gene structure of *DICER* and *PTEN* and the miR-103 recognition sites located in the 3′-untranslated region (3′-UTR) are shown as red rectangles; (**B**) Sequence alignment of miR-103 with reverse complementary miR-103 (rcmiR-103-WT), *DICER* (DICER-3′-UTR-WT), *PTEN* (PTEN-3′-UTR-WT), mutant rcmiR-103 (rcmiR-103-MUT), mutant *DICER* (DICER-3′-UTR-MUT), and mutant *PTEN* (PTEN-3′-UTR-MUT), mutant nucleotides are underlined; and (**C**–**E**) Dual-luciferase reporter assay using constructed vectors alone or in the presence of miR-103 precursor or inhibitor was performed. Vectors contain rcmiR-103-WT and rcmiR-103-MUT was used as controls. *Renilla* luciferase was measured and normalized to Firefly luciferase activity, and the recombinant vector was normalized to empty vector. Data are representative of three experiments. ^a^ *p* < 0.05; ^b^ *p* < 0.01 *vs.* vector alone group.

As we know, miRNAs exert biological functions by regulating their specific target genes. To determine the detail mechanism that miR-103 promotes colorectal cancer, miRNA target prediction algorithms were used to predict target genes of miR-103. *DICER*, an RNase III enzyme which is essential for maturation of miRNAs and implicated as a tumor suppressor [15], and *PTEN*, another canonical tumor suppressor [16], were consequently identified as two putative targets of miR-103 (Figure 3A).

To identify the targeting of *DICER* and *PTEN* by miR-103, dual-luciferase reporter vectors were constructed containing the predicted seed sequences in the 3′-untranslated region (3′-UTR) of *DICER* and *PTEN* (Figure 3B). The empty vector psiCHECK-2 and the vector containing the reverse complementary miR-103 (rcmiR-103) were used as negative and positive controls (Figure 3C) respectively. All these vectors were used to transfect HEK293T cells alone or co-transfect with miR-103 precursor or inhibitor. Results showed that miR-103 precursor apparently repressed, but inhibitor up-regulated, the Rluc containing the seed sequence in the 3′-UTR of *DICER* and *PTEN*. Then, mutations were introduced into the seed sequence to identify whether miR-103 interacted with *DICER* and *PTEN* directly. Results showed that neither miR-103 precursor nor inhibitor affected Rluc activity containing the mutant seed sequence (Figure 3D,E).

**Figure 4.**
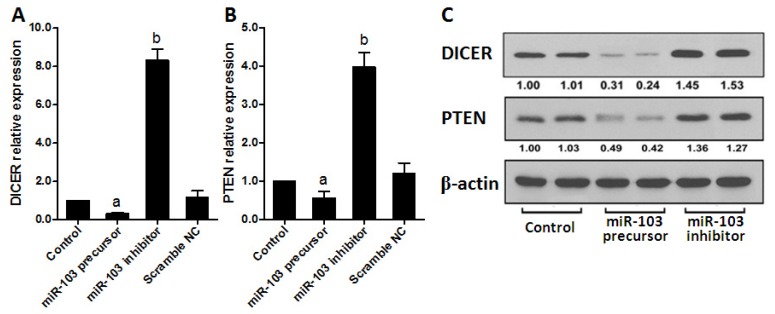
miR-103 regulates *DICER* and *PTEN* expression in colorectal cancer cells. (**A**,**B**) mRNA levels of *DICER* and *PTEN* were determined by real-time PCR in transfected HCT-116 cells; and (**C**) Western blots were used to confirm the expression of *DICER* and *PTEN* in HCT-116 cells after transfection and β-actin was used as control. Data are representative of three experiments. Error bars represent as mean ± SD. ^a^ *p* < 0.05; ^b^ *p* < 0.01 *vs.* control.

To further confirm that miR-103 directly regulates *DICER* and *PTEN* in colorectal cancer cells, both mRNA and protein levels of *DICER* and *PTEN* were determined in HCT-116 cells transfected with miR-103 precursor or inhibitor. As shown in Figure 4A,B, miR-103 precursor dramatically repressed, whereas miR-103 inhibitor increased, *DICER* and *PTEN* expression compared with control. Collectively, all these data demonstrated that *DICER* and *PTEN* are direct targets of miR-103 in colorectal cancer.

### 2.4. miR-103 Promotes Cell Proliferation and Migration by Repressing DICER and PTEN

**Figure 5.**
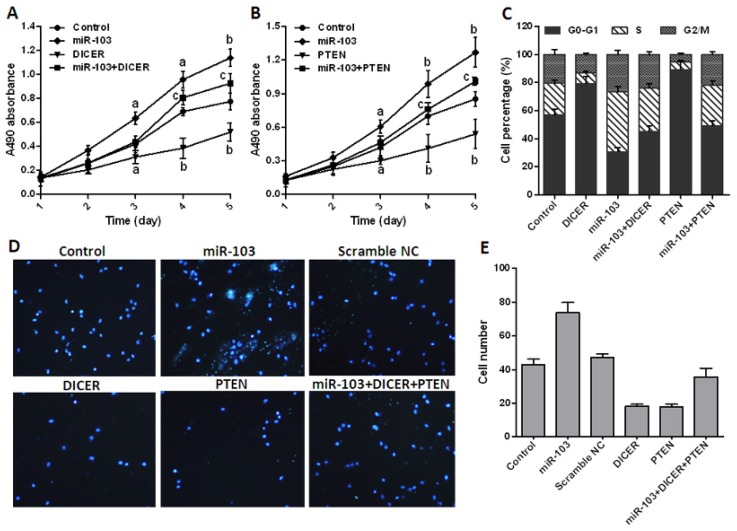
miR-103 promotes colorectal cancer cell proliferation and cell cycle by targeting *DICER* and *PTEN*. (**A**,**B**) 3-(4,5-Dimethylthiazol-2-yl)-2,5-diphenyltetrazolium bromide (MTT) assay was used to check the proliferation rates of transfected HCT-116 cells; (**C**) Cell cycle distributions of transfected HCT-116 were analyzed by flow cytometry; (**D**) Transwell assay was used to detect cell migration (40× magnification); and (**E**) analysis results of **D**. Data are representative of three experiments. Error bars represent as mean ± SD. ^a^ *p* < 0.05; ^b^ *p* < 0.01 *vs.* control; ^c^ *p* < 0.05 *vs.* miR-103 transfection group.

To determine that miR-103 promotes colorectal cancer by regulating *DICER* and *PTEN*, pcDNA-3.1-DICER or pcDNA-3.1-PTEN was transfected into HCT-116 cells alone or in combination with miR-103 precursor. MTT assay showed that miR-103 significantly promoted, but DICER and PTEN inhibited cell proliferation, and both *DICER* and *PTEN* apparently abrogated the effect of miR-103 in co-transfection groups when compared with the control (Figure 5A,B). Flow cytometry results showed that cell cycle was strongly arrested at G0/G1 phase in DICER or PTEN over-expressed cells. In contrast, the proportion of cells was remarkably reduced at G0/G1 phase and increased at S phase in miR-103 precursor alone or combination groups (Figure 5C).

Then, a transwell assay was performed to examine the potential role of *DICER* and *PTEN* in perturbing miR-103 mediated tumor migration. Figure 5D,E showed that HCT-116 transfected with miR-103 precursor exhibited more invasion ability compared with that transfected with pcDNA-3.1-DICER or pcDNA-3.1-PTEN (*p* < 0.01). All these data disclosed that miR-103 effectively promotes colorectal cancer proliferation and migration via repressing *DICER* and *PTEN*.

### 2.5. Repressed miR-103 Expression Inhibits Cancer Cell Growth in Vivo

To evaluate the effect of miR-103 *in vivo*, HCT-116 cells were firstly transfected with Lenti-miR-103 or agomir-miR-103 to up- or down-regulate endogenous miR-103 expression and screened by puromycin. Then xenografts model was generated in five groups of 8 mice each by injecting subcutaneously with HCT-116 cells at a single site. Tumor onset was measured with calipers at the site of injection every three days after injection when appreciable tumor formed subcutaneously. Tumor volume was calculated using the formula, *V* = 0.5ab^2^, where a represents the larger and b represents the smaller of the 2 perpendicular indexes. Animals were sacrificed 5 weeks after injection and tumors were weighed.

**Figure 6.**
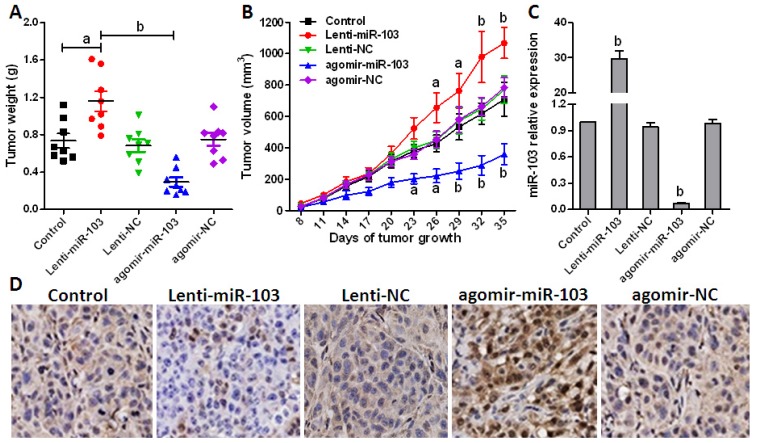
miR-103 promotes colorectal cancer xenograft growth. (**A**) Xenograft models (*n* = 8) in nude mice were generated with transfected HCT-116 cells as indicated. Tumor size was measured every three days for 5 weeks; (**B**) Xenograft tumors in nude mice were weighed; (**C**) Relative miR-103 expression in xenografts was analyzed by real-time PCR; and (**D**) *PTEN* in tumor tissues from various groups was detected with immunohistochemistry using specific *PTEN* antibodies (200× magnification). Error bars represent as mean ± SD. ^a^ *p* < 0.05; ^b^ *p* < 0.01 *vs.* control.

As shown in Figure 6A,B, when compared with NC or miR-103 overexpressed group, the xenografts generated with miR-103 down-regulated cells grew more slowly and had markedly smaller size and lower weight. miR-103 level in the derived xenografts was then measured at d35 post injection, and results indicated that miR-103 expression levels were still stably regulated (Figure 6C). Meanwhile, immunohistochemistry was performed according to an optimized protocol to confirm that DICER and PTEN expression is negatively correlated with miR-103 levels and contributes to miR-103 regulated tumor growth *in vivo* [17] (Figure 6D). Taken together, these results demonstrated that miR-103 functions as an oncogenic miRNA in colorectal cancer cells through decreasing the tumor suppressor genes *DICER* and *PTEN*.

## 3. Experimental Section

### 3.1. Materials

McCoy 5A, RPMI 1640, DMEM and DMEM/F-12 were purchased from Gibco (Grand Island, NY, USA). Restriction endonucleases and T4 DNA ligase were from New England Biolabs (Ipswich, MA, USA). The dual-luciferase reporter system and the empty psiCHECK-2 vector were purchased from Promega (Madison, WI, USA). Lipofectamine 2000 and Trizol reagent were obtained from Invitrogen (Carlsbad, CA, USA). TaqMan microRNA Reverse Transcription kits and TaqMan gene expression assays were from Applied Biosystems (Carlsbad, CA, USA). miR-103 precursors and inhibitors, scramble miRNA negative control were purchased from Ambion (Carlsbad). Lenti-miR-103 and Lenti-NC were from Genechem Biotech (Shanghai, China). Agomir-miR-103 and agomir-NC (miRNA mimics conjugated with cholesterol to make it more stable) were from Ribo Biotech (Guangzhou, China). Primary antibodies for DICER, PTEN and β-actin were purchased from Cell Signaling Technology (Beverly, MA, USA). Nucleotides were synthesized by TaKaRa (Shanghai, China). Transwell chambers were purchased from Costar (Cambridge, MA, USA).

### 3.2. Colorectal Carcinoma Patients and Tissue Specimens

Thirty-five frozen specimens of colorectal cancer tissues, their matched adjacent normal tissues and information about the patients were collected from the First Hospital of Jilin University (Jilin, China) and used for Agilient miRNA microarray or RT-PCR analysis. All subjects were diagnosed and confirmed by pathologist. None of the subjects received any biotherapy or chemotherapy treatments before recruitment to this study. All experiments were reviewed and approved by the ethics committees of the First Hospital of Jilin University.

### 3.3. Cell Culture and Transfection

Human colorectal cancer cell lines HCT-116, HT29, Colo205, SW480 and normal colonic cell line FHC were purchased from National Rodent Laboratory Animal Resource (Shanghai, China). All the cancer cells were cultured in McCoy 5A, RPMI-1640 or DMEM medium containing 100 IU/mL penicillin, 100 μg/mL streptomycin, 20 mM glutamine and 10% heat-inactivated fetal bovine serum (FBS). Normal colon FHC cells were grown in DMEM/F-12 medium with 10% FBS, 10 ng/mL cholera toxin, 5 μg/mL transferrin, 5 μg/mL insulin, 100 ng/mL hydrocortisone and extra 10 mM 4-(2-hydroxyethyl)-1-piperazineëthanesulfonic acid (HEPES). All cells were cultured in a humidified atmosphere of 5% CO_2_ at 37 °C.

For transfection, cells were seeded at 50% confluence and 16 h later, cells were transfected with miR-103 precursor/inhibitor or pcDNA-3.1-*DICER*/*PTEN* plasmids with Lipofectamine 2000 (Invitrogen, Carlsbad, CA, USA) according to the manufacturer’s instruction. The scramble miRNA or pcDNA-3.1 plasmid was used as negative controls. Cells were harvested after 48 h for following experiments.

### 3.4. Cell Growth Assay

Cells were seeded in 96-well plates at 6–8 × 10^3^/well and the surviving fractions were determined at 1, 2, 3, 4, 5 days using 3-(4,5-dimethylthiazol-2-yl)-2,5-diphenyltetrazolium bromide (MTT) assay as previously described [18]. The absorbance was measured with a spectrophotometer (Bio-Rad Laboratories, Hercules, CA, USA) at 570 nm. Each experiment was performed in triplicate [19].

### 3.5. Colony Formation Assay

Cells were trypsinized to single cell suspension and seeded in 6-well plates (500/well), and medium was replaced with fresh medium every three days. After 10 days culture, the medium was removed and cell colonies were stained with crystal violet (0.1% in 20% methanol). Pictures were taken using a digital camera to record the results as described [20].

### 3.6. RNA Isolation and Real-Time PCR (Polymerase Chain Reaction)

Total RNA was extracted from cells or frozen tissues with Trizol and then cDNA was synthesized with TaqMan^®^ MicroRNA Reverse Transcription Kit (Applied biosystems, Carlsbad, CA, USA) according to the manufacturer’s instructions. Aliquots of reaction mixture were used for quantitative PCR with TaqMan^®^ 2× Universal PCR Master Mix (Applied biosystems) by following conditions: initial denaturation at 95 °C for 10 min followed by 40 cycles of 95 °C for 15 s, 60 °C for 1 min and 72 °C for 45 s. All PCR experiments were performed in triplicate.

### 3.7. Western Blot Assay

Cells were lysed in ice-cold cell lysis buffer (50 mM pH 8.0 Tris, 120 mM NaCl, 0.5% NP-40, 50 mM NaF, 1 mM phenylmethylsulfonyl fluoride (PMSF), 20 μM sodium orthovanadate, 1× Protease Inhibitors, 1× Phosphatase Inhibitors). Equal amounts of protein were subjected to SDS-PAGE electrophoresis, then electrotransferred to nitrocellulose membranes. The membranes were incubated with DICER, PTEN or β-actin primary and secondary antibodies, and then the specific immunoreactive proteins were developed by an enhanced chemiluminescence.

### 3.8. Vector Construction and Luciferase Reporter Assays

The dual-luciferase psiCHECK-rcmiR-103-WT, psiCHECK-*DICER*-3′-UTR-WT and psiCHECK-*PTEN*-3′-UTR-WT vectors were constructed by synthesizing the candidate seed sequences in the 3′-UTR of *DICER*, *PTEN* or reverse complementary sequence of miR-103 and inserting the annealing products into the psiCHECK-2 vector. For mutant constructs psiCHECK-rcmiR-103-MUT, psiCHECK-*DICER*-3′-UTR-MUT and psiCHECK-*PTEN*-3′-UTR-MUT, 3–4 bp mutations were introduced into the seed sequences. All plasmids were confirmed by DNA sequencing.

For reporter assays, HEK-293T cells were seeded in 24-well plates (1.5–3 × 10^6^/well) and transfected with 0.8 μg recombinant vectors alone or vectors plus 30 nM precursors or inhibitors with Lipofectamine 2000. Firefly and *Renilla* luciferase activities were measured 24 h later using the dual-luciferase reporter assay system from cell lysates.

### 3.9. Cell Cycle Analysis

HCT-116 cells were seeded in 6-well plates and synchronized in serum free medium for 48 h. After released into the cell cycle by adding 10% FBS to the medium, cells were transfected with scramble miRNA, miR-103 precursor/inhibitor, pcDNA-3.1-*DICER* or pcDNA-3.1-*PTEN* alone or in combination, then harvested, fixed with 70% ethanol, and stained with propidium iodide (PI). The data were acquired by flow cytometry and analyzed using FlowJo software (TreeStar, Cupertino, CA, USA).

### 3.10. Cell Migration and Invasion Assay

Cells were starved in serum-poor Dulbecco’s Modified Eagle’s Medium (Gibco, Grand Island, NY, USA) for 24 h after transfection of 48 h and then performed using 6.5 mm Transwell chambers with 8 μm pores (Costar, Cambridge, MA, USA) as described previously. In brief, the bottom surface of each membrane was coated with fibronectin. Then, HCT-116 cell (1 × 10^5^) suspension was seeded into the upper chambers and 600 μL of complete medium was added into the lower chambers. After incubated at 37 °C for 16 h, the upper surface of each membrane was cleaned with a cotton swab. Cells adhering to the bottom surface of each membrane were stained with 4′,6-diamidino-2-phenylindole (DAPI, Sigma, St. Louis, MO, USA) and counted.

### 3.11. Colorectal Carcinoma Cell Xenografts Growth

Animal experiments were approved by the Institutional Animal Care and Use Committees of Jilin University. Six-week-old female athymic (nu/nu) mice were purchased from Shanghai Slac Laboratory Animal Co., Ltd., Shanghai, China. HCT-116 cells (7 × 10^6^) were transfected with Lenti-miR-103, Lenti-NC, agomir-miR-103 or agomir-NC according to the manufacturer’s protocols, screened for stability, and then were subcutaneously injected into the flank region respectively. Tumor size was measured once every three days and the tumor volumes were calculated as previously described [21]. Mice were sacrificed after 5 weeks and the tumors were removed for immunohistochemistry analysis.

### 3.12. Statistical Analysis

All data were represented as the mean ± SD from at least three independent experiments. Student’s *t*-test for two groups or one-way analysis of variance (ANOVA) and post hoc multiple comparisons (LSD test) for three or more groups were performed to evaluate the statistical significance by using GraphPad InStat 3 software (GraphPad Software, Inc., San Diego, CA, USA). Results were considered statistically significant at *p* < 0.05.

## 4. Conclusions

Despite obvious improvements in cancer therapy during the past few decades, colorectal cancer remains one of the most common diagnosed cancers worldwide and there is an urgent need for the demonstration of the detail mechanisms of colorectal cancer. It is believed that colorectal cancer is usually derived from uncontrolled cell growth in the colon or rectum of the large intestine and most patients with colorectal cancer die of uncontrolled metastatic spread within a few years of diagnosis. Metastasis is a main cause of death in cancer patients and contributes to the high mortality of colorectal cancer. However, the mechanism of colorectal cancer cell growth and metastasis remains unclear.

The finding of miRNAs has greatly accelerated our understanding of the mechanisms that regulate genes expression. By binding to the 3′-UTR of their target genes, miRNAs can post-transcriptionally modulate genes expression by repressing translation or accelerating mRNA degradation. Increasing evidence demonstrated that miRNAs play important roles in various physiological and pathological processes including developmental abnormalities, autoimmune diseases and cancers [22,23].

Several studies have shown that miR-103 is involved in various biological and pathological processes. Trajkovski has reported that miRNA-103 is up-regulated in obese mice and silencing of miR-103 leads to improved glucose homeostasis and insulin sensitivity [24]. A recent report showed that miR-103, together with miR-142-3p, miR-30b and miR-342-3p were significantly down-regulated in heart failure (HF) and all these miRNAs might be used as a marker for HF diagnosis [25]. Not surprisingly, miR-103 is also reported to associate with several human cancers. Such as miR-103 can post-transcriptionally down-regulate the expression of the tumor suppressor gene tissue inhibitor of metalloproteinase 3 (TIMP-3) and promote growth and invasion of endometrial cancer cell lines [26]. Although it has been reported recently that miR-103 was expressed in colorectal cancer as an oncogenic miRNA by targeting *DAPK*, *KLF4* and *PER3* [27,28], the detail mechanism of miR-103 in colorectal cancer growth and metastasis is still largely unknown.

In this study, we demonstrated that miR-103 is up-regulated in colorectal cancer and significantly promoted cancer cell proliferation, invasion and metastasis *in vitro* and *in vivo*. Further studies identified that *DICER* and *PTEN*, two critical molecules function as tumor suppressors, as targets of miR-103. Consistently, down-regulation of miR-103 or ectopic expression of *DICER* and *PTEN* apparently inhibited HCT-116 cancer cell growth and migration. Down-regulation of miR-103 by agomir-miR-103 transfection significantly inhibited colorectal cancer cell growth in xenograft models. All these findings indicate that miR-103 plays a critical role in colorectal carcinogenesis.

*DICER* and *PTEN* are two critical tumor suppressor genes. It was reported that up-regulated miR-20b, miR-21 and miR-130b in colorectal cancer can inhibit the expression of *PTEN* [29]. We report here that *DICER* and *PTEN* are direct target genes of miR-103, and thus, dramatically down-regulated by miR-103 at the posttranscriptional level in colorectal cancer. Up-regulation of these two genes or inhibiting miR-103 expression resulted in a significant reduction of cancer cell proliferation and migration *in vitro* and tumor growth *in vivo*. Our work broadens the understanding of the roles that miR-103 plays in colorectal cancer by targeting several different genes, and thus can be a more powerful target for cancer therapy. In addition, our data shed light on the possibility of fine tuning of target genes such as *PTEN* by a group of miRNAs in human cancer.

In summary, we demonstrate that miR-103 acts as an oncogene miRNA in colorectal cancer. Through regulation of colorectal cancer cell *DICER* and *PTEN* expression, miR-103 significantly promotes cancer cell proliferation, invasion and metastasis, and, thus, could be an important mediator in the pathogenesis of colorectal cancer. Manipulation of miR-103 axis expression represents a novel potential therapeutic target for colorectal cancer treatment.
